# Textural analysis of late gadolinium enhanced magnetic resonance images can discriminate acute from chronic myocardial infarction

**DOI:** 10.1186/1532-429X-16-S1-P182

**Published:** 2014-01-16

**Authors:** Pascale Beliveau, Farida Cheriet, Stasia A Anderson, Joni Taylor, Andrew E Arai, Li-Yueh Hsu

**Affiliations:** 1 National Heart Lung and Blood Institute, National Institutes of Health, Rockville, Maryland, USA; 2 Ecole Polytechnique of Montreal, Montreal, Quebec, Canada

## Background

Late gadolinium enhanced magnetic resonance (LGE MR) imaging is the current standard modality for evaluating the extent of myocardial damage in the presence of ischemic cardiomyopathies (ICM). However it is difficult to differentiate acute from chronic myocardial infarctions (MI) by standard signal intensity thresholding methods. We hypothesize that textural analysis of myocardial infarct regions has the potential to discriminate acute from chronic MI in high resolution LGE CMR images.

## Methods

The experiment was performed on 10 rats (8 month-old males). Five acute MI were created with 30-minute LAD occlusion and imaging was performed 3 hours post-reperfusion. Permanent ligature of the LAD was performed for the 5 chronic MI rats, imaged 2 months after the intervention. LGE MR images were acquired at 50-55 μm isotropic voxel resolution using a 3D gradient echo sequence on a 7T Bruker scanner. Histological staining on short-axis slices of the excised hearts (myoglobin for acute and Masson Trichrome for chronic MI) was the reference for manual segmentation of infarct regions of interest. Three texture features (homogeneity, energy and entropy) were derived from Haralick's grey level co-occurrence matrix and computed in 3D. Student's T-test was performed to compare the two groups.

## Results

**Figure 1:**
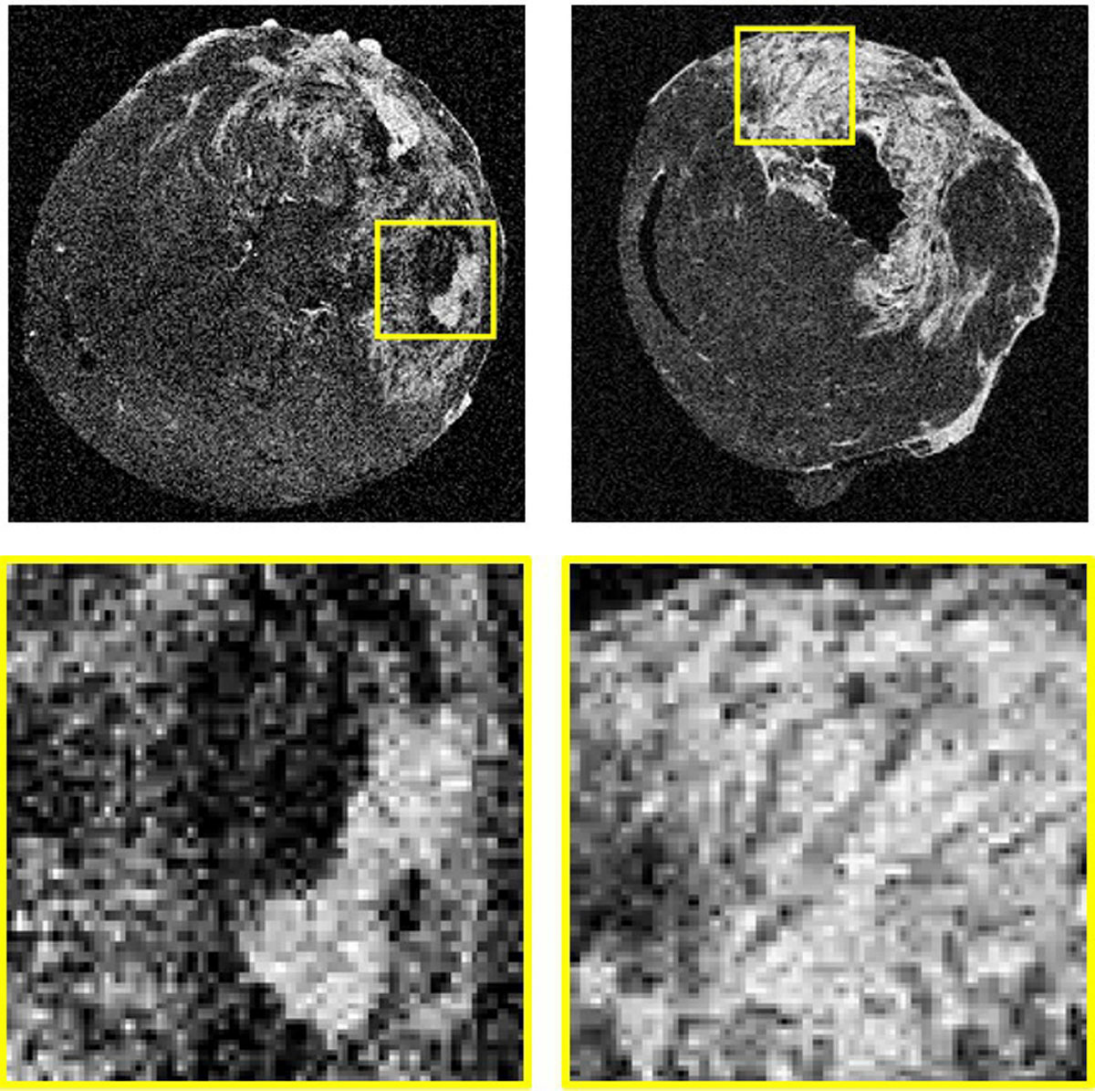
**Texture analysis discriminates acute (left panel) vs. chronic (right panel) myocardial infarcts in high resolution LGE CMR images of ex-vivo rat heats**. A higher textural complexity can be explained by the presence of microvascular obstructions in the acute infarct (bottom left) compared to a more confluent texture in the chronic infarct (bottom right).

All texture features were significantly different between acute versus chronic MI (p < 0.05). The homogeneity and energy texture values were 1.40 and 1.43 times higher in chronic than in acute MI. A higher homogeneity or energy value indicates a flatter pixel-to-pixel variation. However, the entropy texture was 1.12 times higher in acute MI which indicates a textural complexity in the infarct regions by the presence of microvascular obstructions. Figure 1 shows example images of acute and chronic MI for the ex vivo rat hearts.

## Conclusions

Textural features were significantly different in animal models of acute vs. chronic MI with high resolution LGE CMR imaging. Further study is needed to establish the performance of discriminating MI age with texture features in a clinical setting. Textural analysis, a reproducible method, of myocardial infarct regions in LGE CMR images could potentially improve the quantification and diagnosis of MI in ischemic cardiomyopathies.

## Funding

This research was supported by the Intramural Research Program of the National Heart, Lung, and Blood Institute, National Institutes of Health.

